# Small extracellular vesicles in breast cancer brain metastasis and the prospect of clinical application

**DOI:** 10.3389/fbioe.2023.1162089

**Published:** 2023-04-05

**Authors:** Tingli Luo, Yue Kang, Yushi Liu, Jingyue Li, Jianyi Li

**Affiliations:** Department of Breast Surgery, Liaoning Cancer Hospital, Shenyang, China

**Keywords:** exosomes, breast cancer, brain metastasis, blood-brain barrier, liquid biopsy, extracellular vesicles

## Abstract

Extracellular vesicles (EVs) are nanoscale extracellular particles that have received widespread scientific attention for carrying a variety of biomolecules such as nucleic acids and proteins and participating in the process of intercellular information exchange, making them become a research hotspot due to their potential diagnostic value. Breast cancer is the leading cause of cancer-related death in women, approximately 90% of patient deaths are due to metastasis complications. Brain metastasis is an important cause of mortality in breast cancer patients, about 10–15% of breast cancer patients will develop brain metastasis. Therefore, early prevention of brain metastasis and the development of new treatments are crucial. Small EVs have been discovered to be involved in the entire process of breast cancer brain metastasis (BCBM), playing an important role in driving organ-specific metastasis, forming pre-metastatic niches, disrupting the blood-brain barrier, and promoting metastatic tumor cell proliferation. We summarize the mechanisms of small EVs in the aforementioned pathological processes at the cellular and molecular levels, and anticipate their potential applications in the treatment of breast cancer brain metastasis, with the hope of providing new ideas for the precise treatment of breast cancer brain metastasis.

## 1 Introduction

**FIGURE 1:**
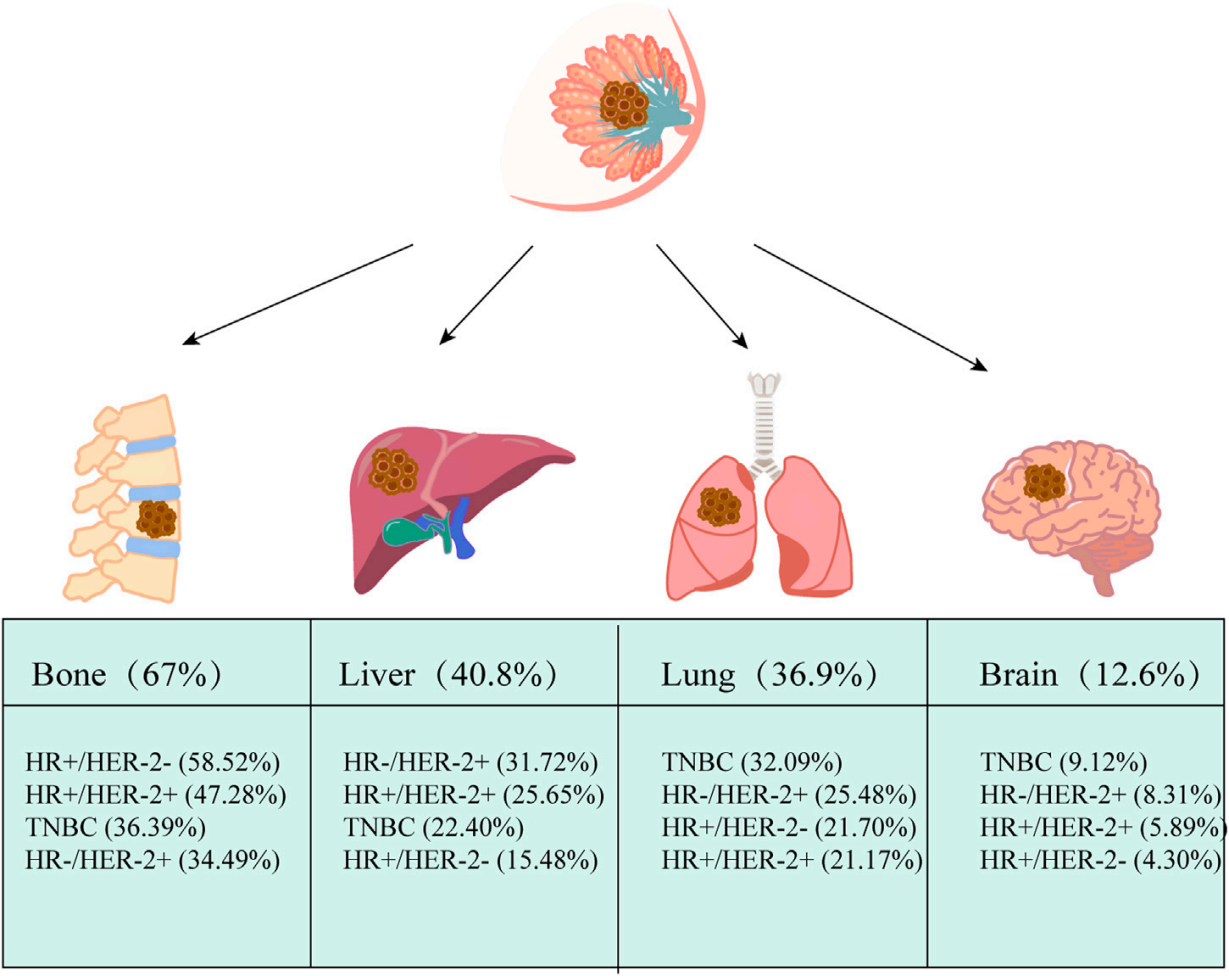
Common distant metastasis in breast cancer. The most common distant metastatic organ for breast cancer is the bone, with the liver coming in second, followed by the lung and brain. Based on hormone receptor (HR) and human epidermal growth factor receptor 2 (HER2) status, breast cancer was classified into four subtypes: hormone receptor (HR) +/human epidermal growth factor receptor 2 (HER2) −, HR+/HER2+, HR−/HER2+ and triple-negative (TN). Based on data from Surveillance, Epidemiology, and End Results Program (SEER) (2010–2013), The pathologic subtypes of 243,896 invasive breast cancers differed markedly in metastatic behavior at distant metastatic sites, with triple-negative breast cancer (TNBC) and HR+/HER2+ being more likely to have brain metastases. The data comes from (Wu et al., 2017).

According to the latest global cancer statistics data released by International Agency for Research on *Cancer* (IARC), the number of new breast cancer cases worldwide has reached 2.26 million in 2020, surpassing lung cancer as the world’s most common cancer. Breast cancer is the leading cause of death in females among malignant tumors (Sung et al., 2021). Approximately 90% of patients die due to metastasis complications (Chaffer and Weinberg, 2011). Common distant metastatic organs for breast cancer include bone, brain, liver, lung, and different subtypes of breast cancer have distinct tropisms (Buonomo et al., 2017). For instance, triple-negative breast cancer (TNBC) and HER2-positive are the high-risk subtypes of brain metastasis (Darlix et al., 2019) (Figure 1). Although the incidence of BCBM, 10–15%, is lower than in other metastatic organs, there was a high mortality rate and a low survival rate with a median survival of only 10 months (Martin et al., 2017), and the efficacy of existing treatments is unsatisfactory. As a result, new preventive and therapeutic tools are needed urgently.

Recent research has reported that small EVs have a central role in the entire process of BCBM. This review summarized the mechanisms of small EVs in the processes and anticipates their potential application in the treatment of BCBM, aiming to provide new ideas for the precise treatment of BCBM.

## 2 Exosome and extracellular vesicles

PAN et al. discovered exosomes in the 1980s, and after the discovery in the 1990s that B lymphocytes-secreted exosomes could present antigens to activate T-lymphocytes and induce immune responses, exosomes have received widespread attention from scientists. (Pan and Johnstone, 1983; Johnstone et al., 1987). Over the last decades, a large number of articles have demonstrated that exosomes exert an important role in physiological processes such as aging, cancer, and obesity (Corrado et al., 2013; Lei et al., 2021).

The term exosomes were adapted to define nanovesicles of endosomal source that are liberated by the fusion of multivesicular body (MVB) with the plasma membrane (Johnstone, 1992). However, owing to inconsistencies in laboratory purification methods and the inability to fully purify to a specific “exosome” population, the definition of " exosomes " (e.g., diameter range) varies across many articles, not fully standardized.

**FIGURE 2:**
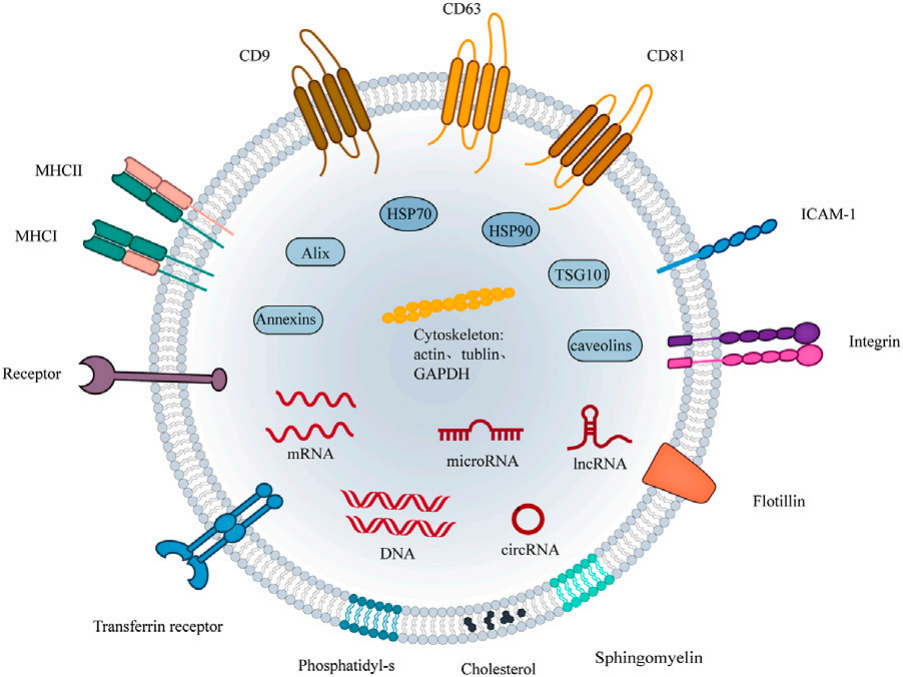
The structure of small EVs. The components of small EVs consist of three main groups of substances. (1) Proteins: four transmembrane protein superfamily: CD9, CD81, CD63, etc; Heat shock proteins: HSP70 and HSP90; Cytoskeletal proteins: actin, tubulin, etc; Adhesion proteins: ICAM1, integrins and transferrin; Antigen presentation: MHC-Ⅰ, MHC-Ⅱ,CD86. Among them, CD9, CD81, CD63, flotillin, TSG101, ceramide, and Alix are currently considered markers of small EVs. (2) Nucleic acids: DNA, mRNA, miRNA, and other non-coding RNAs. (3) Lipids: cholesterol, sphingomyelin, phosphatidyl-s, *etc.*

The International Society for Extracellular Vesicles (ISEV) has defined the term “extracellular vesicles (EVs)“ and defined EVs subtypes based on the physical characteristics of EVs, replacing “exosomes” and “microvesicles” to enable the early realization of the value of EVs as biomarkers or therapeutic applications. The term extracellular vesicle refers to natural particles released from the cell and delimited by a lipid bilayer and cannot self-replicate. Guidelines set by the ISEV2018 differentiate EVs subtypes based on physical characteristics such as EV size (small EVs<100 nm or <200 nm, or medium/large>200 nm), density, and biochemical composition (e.g. CD63, CD81, Annexin A5) (Théry et al., 2018). Tetraspanins (e.g. CD9, CD63, CD81), MVB biogenesis-related proteins (Alix, and TSG101), and heat-shock proteins are generally small EVs-specific proteins (An et al., 2015; Théry et al., 2018; Kalluri and LeBleu, 2020) (Figure 2).

This review will concentrate on small EVs and references dealing with “exosomes” will fall into this category as suggested by the ISEV. EVs can be produced and released by almost all cell types and are widely found in body fluids such as blood, saliva, and urine (Pisitkun et al., 2004; Sharma et al., 2010; Zomer et al., 2015). Small EVs contain diverse cargo including proteins, lipids, and nucleic acids (DNA, mRNA, microRNAs, and non-coding RNAs), which act as mediators of intercellular communication (Valadi et al., 2007; Kosaka et al., 2010). Studies have demonstrated that, especially in hypoxic environments, tumor cells can generate more EVs than normal cells (Park et al., 2010; Bebelman et al., 2018). Small EVs participate in the processes of tumorigenesis, proliferation, metastasis, and drug resistance formation by carrying different components (Sundararajan et al., 2018; Dong et al., 2020).

## 3 Small EVs mediated brain metastasis in breast cancer

### 3.1 Organ-specific metastasis and the formation of pre-metastatic niches

**FIGURE 3:**
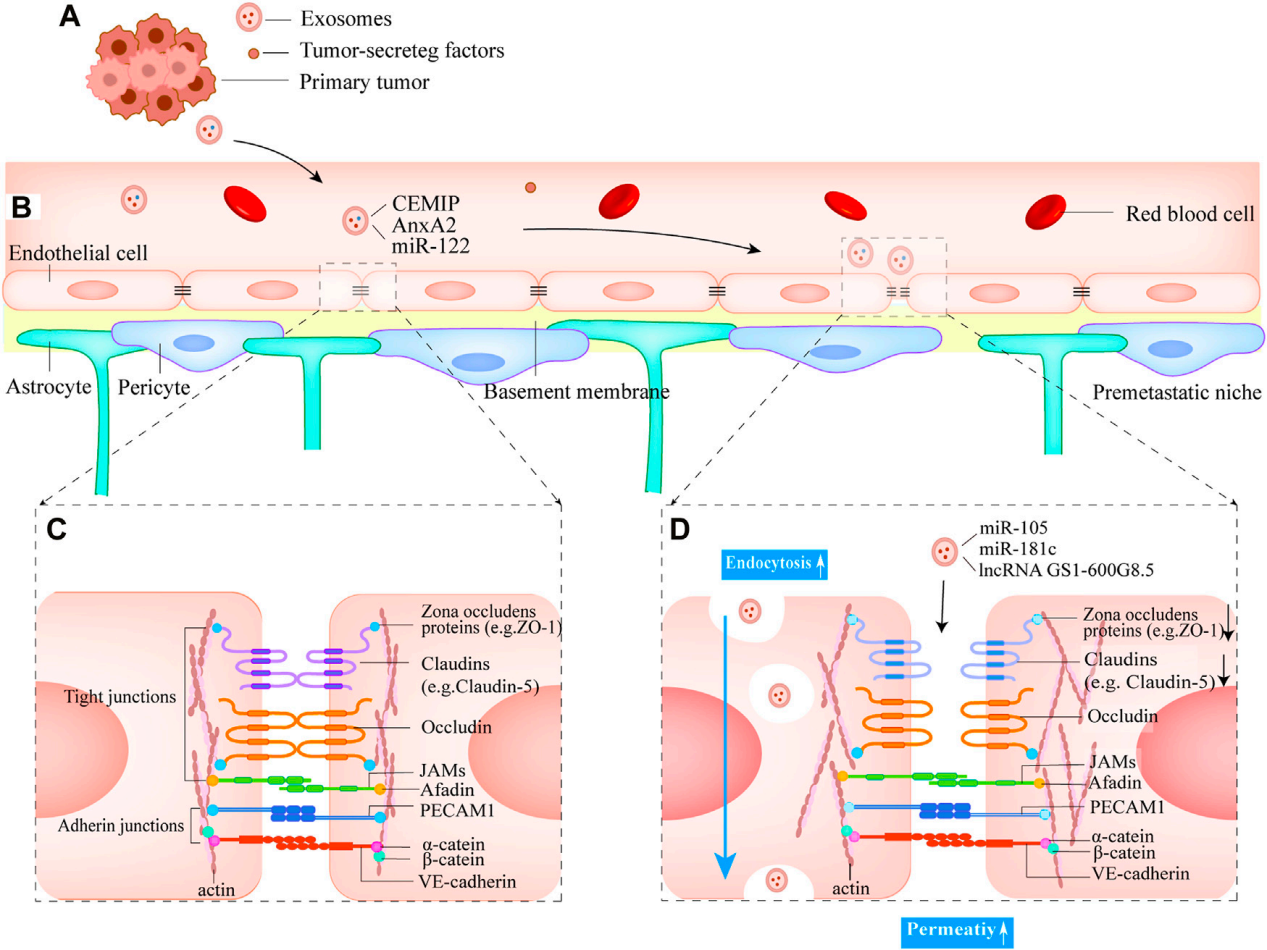
Small EVs mediate the formation of pre-metastatic niches and the destruction of the BBB **(A)** Breast tumor cells release small EVs into the blood and eventually form the pre-metastatic niches in the brain **(B)** Normal structure of BBB **(C)** The structure of the junctional complexes between endothelial cells. The complex type mainly consists of tight junctions and adherens junctions, which can effectively ensure the low permeability of BBB **(D)** Small EVs derived from breast primary tumor cells disrupt tight junctions. Mainly decreases the expression of proteins ZO-1 and Claudin 5 and increases BBB permeability. Small EVs cross the BBB by endocytosis.

The organ-preference patterns of tumor metastasis have been the greatest puzzle since Stephen Paget proposed the “seed and soil” hypothesis in 1889 (Paget, 1989). For instance, lung cancer, breast cancer, and melanoma are the most frequent metastasize to the brain (Suh et al., 2020). Recent studies have uncovered that small EVs have a significant impact on this process. The primary tumor site can create favorable microenvironments in secondary organs termed pre-metastatic niches by pre-releasing small EVs that are conducive to tumor cell growth before tumor cell distant metastasis occurs. (Guo et al., 2019) (Figure 3A).

For instance, Hoshino et al. (2015) showed that small EV-derived integrins (ITGs) were strongly related to organ-directed metastasis by carrying out a proteomic analysis of small EVs. Respectively, ITGβ4 and ITGβ3 present in small EVs derived from breast cancer cells specifically mediate lung and brain metastasis. Moreover, the results were confirmed in serum samples from patients with lung metastasis, and ITGβ4 has the clinical potential to predict lung metastasis in breast cancer patients. However, the patient’s serum sample did not demonstrate ITGβ3 to mediate specific transfer of small EVs to the brain. A subsequent study by Rodrigues et al. (2019) found that cell migration-inducing and hyaluronan-binding protein (CEMIP), a Wnt-related protein, was highly enriched brain metastatic tumor-derived small EVs and demonstrated that CEMIP can promote brain pre-metastasic niches and cancer cell colonization by upregulating the expression of a series of cytokines. The results of the group suggest that CEMIP can predict the progression of BCBM and patient survival and that targeting small EVs CEMIP holds promise as a potential means of preventing and treating breast cancer brain metastasis.

In addition to the aforementioned studies, Maji et al. (2017) gained insight from the data of proteomic analysis of Exocarta and Jeon that Annexin A2 (AnxA2), a protein associated with processes such as fibrinogen activation, actin-cytoskeleton rearrangement, and cell migration, is abundant in small EVs and that AnxA2 levels were positively correlated with the invasiveness of breast cancer cells. They further demonstrated that AnxA2 carried by small EVs could promote more brain metastasis (∼4-fold), activate p38 MAPK, NF-κB, and STAT3 pathways in macrophages, increase secretion of IL-6 and TNF-α and establish the pre-metastatic sites in the brain, using an intracardiac injection of a small EVs mouse model. Small EVs contain a large number of proteins, and variations in protein expression are critical characteristics for identifying diverse small EVs sources and destinations (Mathivanan et al., 2010).

MicroRNAs (miRNAs) found in small EVs, in addition to proteins, have been shown to play an important role in the formation of pre-metastatic niches. The Warburg effect is known to be widespread in tumor cells with low adenosine-triphosphate (ATP) production efficiency (Vander Heiden et al., 2009). To meet the demands of their rapid growth, cancer cells tend to increase their glucose uptake in a variety of ways. In addition to increasing glucose transporters (GLUT1) and glycolysis-related enzymes (Kang et al., 2002), cancer cells can obtain more glucose from the blood by increasing angiogenesis. When tumor cells spread to target organs and are ready to obtain nutrients quickly in these ways, surrounding cells become their nutrient competitors, which is unquestionably harmful to tumor cells. As a result, inhibiting these competitors’ glucose uptake before metastasis becomes a critical means for the subsequent rapid proliferation of tumor cells. Fong et al. (2015) discovered that breast cancer cells can inhibit non-tumor cell glucose uptake by secreting small EVs containing high levels of miR-122 and downregulating pyruvate kinase expression in the brain, confirming that small EVs can increase proliferation and metastasis by altering glucose metabolism at an early stage, making more glucose available for future metastatic cancer cells.

### 3.2 Breast cancer cell-secreted small EVs facilitate tumor cell extravasation across the blood-brain barrier

The blood-brain barrier (BBB) is a highly complex and dynamic central nervous system structure, mainly composed of brain microvascular endothelial cells (BMECs), pericytes, basement membranes, and astrocytes (Cd et al., 2020) (Figure 3B). BMECs are the most abundant cellular component of the BBB. Compare to other vascular endothelial cells, BMECs have abundant tight junctional proteins (TJs) and extremely weak endocytosis, which can strictly limit the entry of various substances into the brain and contribute to the physical barrier between the peripheral circulatory system and central nervous system (CNS) (Banks, 2009; Chow and Gu, 2015).

Since the lack of classical lymphatic circulation in the central nervous system, the blood-borne route is critical for tumor cells that intend to metastasize to the brain parenchyma and must breach the BBB. (Louveau et al., 2015; Da Mesquita et al., 2018). However, the precise mechanism of how tumor cells successfully cross the BBB remains unknown. In contrast to breast cancer metastasizing to other organs, the key step with brain metastasis is the various interaction with the cellular components of the BBB. *Cancer* cells secrete humoral factors during this event, causing BBB destruction and tumor cell extravasation (Li et al., 2017; Curtaz et al., 2020). Similarly, tumor-derived small EVs have been shown in studies to participate in the above process, creating favorable conditions for subsequent tumor cells to pass through the BBB.

#### 3.2.1 Promote the adhesion of tumor cells to endothelial cells by breast cancer cell-secreted small EVs

Studies in animal models of brain metastasis have shown that circulating metastatic cancer cells first adhere to BMECs before passing through the BBB, which is a critical event (Kienast et al., 2010; Xiao et al., 2020). Tumor cell-derived small EVs have been shown to have a pre-emptive effect on brain endothelial cells, influencing the adhesion kinetics between subsequent tumor cells and endothelial cells. The cytoskeleton-associated protein Tubulin Tyrosine Ligase Like 4 (TTLL4) overexpression in breast cancer cells is associated with brain metastasis and alters small EVs biogenesis. Upregulation of TTLL4 in breast cancer cells promotes small EVs secretion, which increases the permeability of BBB endothelial cells and the adhesion of TNBC to endothelial cells, allowing tumor cells to pass through the BBB more easily. Further research is needed to determine whether this process involves the upregulation of Inter-cellular adhesion molecule 1 (ICAM-1) expression. TTLL4 could be a promising therapeutic target. (Tamura et al., 2020).

#### 3.2.2 Disrupted tight junctions by breast cancer cell-secreted small EVs

Tight junction complexes between BMECs are abundant and are the main contributors to BBB barrier function (Liu et al., 2012; Meena et al., 2022). They are primarily composed of proteins such as transmembrane tight junction proteins claudins (e.g., claudin-5), occludin, and cytoplasmic proteins zonula occludens (e.g., ZO-1, ZO-2) (Chow and Gu, 2015) (Figure 3C). Small EVs from metastatic breast cancer cells contain miR-105, which reduces the expression of ZO-1, resulting in intercellular tight junction disruption and increased BBB permeability (Figure 3D). Furthermore, serum miR-105 levels serve as a predictor of breast cancer metastasis (Zhou et al., 2014)**.**

Actin is a cytoskeleton component that is required for the formation of cell protrusions that are involved in adhesion, chemotaxis (filamentous pseudopods), migration (lamellar pseudopods), and invasion (invasive pseudopods) (Dugina et al., 2016). The major cytoskeletal protein, actin, has known binding sites on all ZO proteins, claudins, and Occludin (Van Itallie et al., 2017; Brunner et al., 2022). In vitro experiments, (N et al., 2015), discovered that breast cancer cells transfer miR-181c into endothelial cells by secreting small EVs that inhibit the target gene 3-phosphoinositide-dependent protein kinase 1 (PDPK1), resulting in the downregulation of phosphorylated cofilin and the resultant activated cofilin-induced modulation of actin dynamics. Finally, the tight junction proteins that were originally expressed on the cell membrane are found in the cytoplasm, and the tight junction complexes’ structure is disrupted. Notably, the tight junction protein expression level was not reduced during this process. It remains to be seen, however, whether miR-181c can be used as a prognostic indicator for patients in the early stages before brain metastasis occurs. Lu et al. (2020) demonstrated that small EVs derived from brain-metastatic breast cancer cells decrease the expression of tight junction proteins (ZO-1, Claudin-5) between BMECs by transporting lncRNA GS1-600G8.5, increasing BBB permeability. However, the downstream targets of lncRNA GS1-600G8.5 are unknown. These studies demonstrate the significance of decreased tight junction protein expression levels caused by small EVs inclusions in BBB disruption and are expected to be a predictor of breast cancer brain metastasis (Figure 3D).

#### 3.2.3 Enhanced BMECs endocytosis by breast cancer cell-secreted small EVs

Cells can internalize small EVs *via* various pathways, including non-specific and receptor-mediated pathways (Sprowls et al., 2019). While most studies have focused on tight junction disruption, Morad et al. (2019) demonstrated that breast cancer cell-derived small EVs do not “squeeze” into the brain from the intercellular space, but rather cross the intact BBB barrier *via* endocytosis transport. Morad et al. (2020) observed that small EVs are endocytosed by continuing astrocytes after crossing the BBB barrier and investigated the mechanism, discovering that small EVs encapsulating miR-301a-3p can be internalized by astrocytes *via* a specific Cdc42-dependent clathrin-independent carrier/GPI-AP-enriched compartment (CLIC/GEEC) endocytic pathway, downregulating the target gene TIMP-2, a matrix metalloproteinase inhibitor. This causes changes in the brain microenvironment, leading to the formation of an ecological niche favorable to tumor cell growth. These studies discover small EVs -driven mechanisms of transport across the BBB in breast cancer brain metastasis, which can be used to develop effective drug delivery methods and early intervention for breast cancer brain metastasis.

### 3.3 Promotes the proliferation of metastatic tumor cells by small EVs

Successful extravasation of metastatic cells to distant metastatic sites often results in a dynamic cross-talk with the metastatic site microenvironment, altering resident cell gene expression patterns to facilitate their proliferation in a microenvironment significantly different from the primary organ (Joyce and Pollard, 2009). Small EVs have also been shown to reshape the brain microenvironment, favoring cancer cell colonization and proliferation. Small EVs from astrocytes mediated the transfer of miRNA-19a targeting PTEN into metastatic breast cancer cells, resulting in a lack of PTEN expression and increased secretion of the cytokine chemokine (C-C motif) ligand 2 (CCL2) as well as recruitment of IBA1-expressing positive myeloid cells to the metastatic site. These myeloid cells aided brain metastatic tumor cells by increasing proliferation and inhibiting apoptosis (Zhang et al., 2015). These findings suggest that astrocyte-derived small EVs can promote the transfer of microRNAs, thereby promoting the proliferation of metastatic breast cancer cells.

**FIGURE 4:**
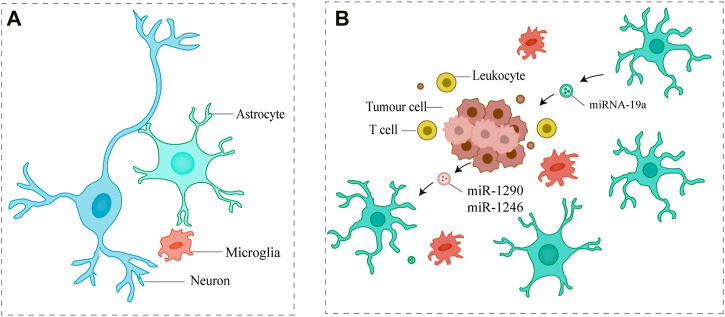
Small EVs promote the proliferation of metastatic tumor cells **(A)** Major cell types in the brain **(B)** Metastatic tumor cells communicate with astrocytes *via* small EVs, which ultimately promote metastatic tumor cell proliferation.

Sirkisoon et al. (2022) demonstrated that breast cancer EV-derived miR-1290 and miR-1246 activate astrocytes in the brain metastatic microenvironment and that EV-derived miR-1290 enhanced intracranial colonization and growth of breast cancer cells, promotes the progression of brain metastases. Sharma et al. (2019) demonstrated that miR-1246 within small EVs secreted by brain metastatic cells can promote tumor angiogenesis. And miR-1246 can be used as a potential biomarker for liquid biopsy (Figure 4B).

## 4 Potential clinical applications of small EVs in BCBM

### 4.1 Small EVs application for liquid biopsy

**FIGURE 5:**
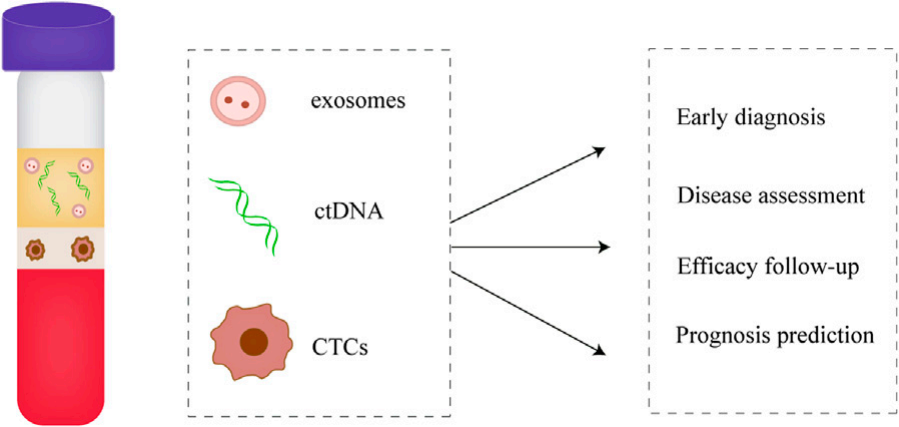
Liquid biopsy tools and the clinical application.

Most brain metastatic lesions are difficult to detect early on. Imaging and cytological examinations are currently the primary tools for confirming brain metastasis. However, imaging examinations frequently lag and cannot reflect the exact changes of the tumor at the same time (Gupta et al., 2010; Butowski, 2015). Furthermore, due to the unique structure of the brain, brain tissue biopsy is frequently only possible during surgery, and the surgical procedure is complicated and prone to postoperative complications for tumors in unusual locations, not to mention the inability to monitor tumor changes dynamically (Patel et al., 2014). When compared to traditional biopsy, liquid biopsy can detect the patient’s overall condition, whereas tissue biopsy can only reflect the information in the tissue sample; a liquid biopsy is a non-invasive operation that can be performed continuously at various stages of the disease, with higher sensitivity and acceptability, and fewer complications (Figure 4.) (Siravegna et al., 2017). As a result, the use of liquid biopsy in central nervous system tumors has numerous advantages. Through *in vitro* non-invasive blood sampling, liquid biopsy obtains circulating tumor cells (CTCs), circulating tumor DNA (ctDNA), small EVs, and other tumor tissues shed in circulating blood, the information of which provides a strong basis for early diagnosis, disease assessment, efficacy follow-up, and prognosis prediction of tumor patients (Fici, 2019) (Figure 5). CTC and ctDNA are now commonly used in clinical practice.

Small EVs have distinct advantages over CTC and ctDNA. For starters, they have a broad origin, all cells can produce small EVs, and they are widely distributed in various body fluids in the human body. In 1 mL of blood, 10^12 small EVs are typically present. In comparison, 1 mL of blood contains only 1–10 CTCs. Second, small EVs have a bilayer lipid membrane structure, are stable in peripheral body fluids (Turchinovich et al., 2011), and are secreted into the blood at the early stage of tumor metastasis, allowing them to be used to predict and diagnose brain metastasis. CTC, on the other hand, has few cells in early tumors or disease, making it difficult to detect, and its main application is in disease evaluation and prognosis (Xie et al., 2020). For instance, CTC greater than or equal to 1 cell/7.5 mL in patients with early-stage breast cancer indicates a poor prognosis (Goodman et al., 2018). Third, because small EVs are derived from living cells, they can more accurately and completely reflect the genetic information of parental cells. Because tumor cells and related immune cells, among others, can produce small EVs, small EVs can reflect a variety of information, including tumor microenvironment (Noerholm et al., 2012; Jeppesen et al., 2019). Small EVs-derived membrane proteins and the biomolecules they contain are important components of tumor-specific biomarkers. According to the previous study, the brain metastatic small EVs CEMIP was highly enriched in small EVs of serum origin from patients surviving brain metastasis from breast cancer (Rodrigues et al., 2019), and AnxA2 was positively correlated with the invasiveness of breast cancer cells, implying that these two types of proteins could be tumor biomarkers (Maji et al., 2017). (Sharma et al., 2019)demonstrated that small EVs miR-1246 could be used as a liquid biopsy biomarker to assess the role of breast cancer-specific amplitude-modulated radiofrequency electromagnetic fields (BCF), a novel treatment for brain metastasis which led to significant and durable regression of brain metastasis of a patient with TNBC. Small EVs, as intercellular information exchange carriers, have demonstrated great promise in basic laboratory research for clinical tumor diagnosis and treatment.

### 4.2 Small EVs as a new target and therapeutic tool

Small EVs production, transport, and uptake are a continuous set of processes. Tumor metastasis involves complex processes such as local invasion, intravasation, and extravasation. Small EVs can affect each step in this cascade reaction, making them a potential target for brain metastasis treatment. Small EVs can be used as a therapeutic target in these ways: inhibiting small EVs production, blocking small EVs uptake, and using small EVs to deliver drugs. Small EVs’ release must be precisely regulated to achieve intercellular communication. Small EVs can be inhibited by suppressing genes associated with small EVs production, based on the currently known pathways of small EVs production (Rab27a, rab27b, rab11a, *etc.*) (Kalluri and LeBleu, 2020; Bai et al., 2022). Using a lentiviral vector, (Naik et al., 2019), demonstrated that ATP9A, a phospholipid-flipping enzyme, inhibits small EVs secretion in MCF-7 breast cancer cells. The exact mechanism of small EVs production is still unknown, The consensus view holds that Rab27a, Alix, and CD63 are required for small EVs biogenesis; however, Gould’s team discovered that knocking out Rab27a, Alix, or CD63 did not affect small EVs biogenesis in relevant experiments (Fordjour et al., 2022). So this therapeutic route needs to be researched further.

Inhibiting small EVs uptake by receptor cells is another strategy for small EVs -targeted therapy. Unfortunately, few molecules have been identified as essential for small EVs uptake by recipient cells to date. The discovery of these molecules could lead to the development of high-specificity, low-side-effect targeted therapies against cancer cell-derived small EVs(Tamura et al., 2020)**.** Furthermore, these molecules may aid in small EVs-mediated drug delivery (Jayasinghe et al., 2022)**.** It is worth noting that small EVs may not be good therapeutic targets against tumor invasion due to the diversity and fragility of the mechanisms by which they mediate tumor cell passage through the BBB.

Chemotherapeutic medications are unable to efficiently enter the brain due to the BBB’s low permeability and the presence of numerous drug-resistance proteins (BCRP, P-gp, *etc.*), which limits their effectiveness in treating patients with brain cancer (Cd et al., 2020). Small EVs can be used to deliver therapeutic medications and biomolecules to the brain since they are only nanoscale in size, are natural carriers of biomolecules, have good tumor-homing capabilities, have great circulating stability, and are not immunogenic. Yang et al. showed that anticancer medicines can pass the BBB through receptor-mediated endocytosis when they are delivered using small EVs (Yang et al., 2015). Small EVs can be uptake by astrocytes and endothelial cells through endocytosis, as demonstrated by Morad et al. (Morad et al., 2019). Due to their endogenous makeup and nanoscale size, small EVs-based chemotherapeutic administration is also believed to increase cytotoxicity, blood circulation time, tumor site accumulation, and drug stability. Targeting specific organs is an important prerequisite for the use of small EVs in brain metastasis therapy, and the cellular uptake and target organ homing potential of small EVs is enhanced with the presence of vesicle surface proteins. Small EVs can be used as a platform for targeted drug administration, but certain naturally occurring small EVs have poor targeting. This can be improved by choosing particular small EVs donors or using bioengineering methods (Jiang et al., 2022). Engineering modifications can greatly improve the targeting ability of small EVs-derived surface proteins and other proteins, improving the therapeutic application of small EVs and increasing their effectiveness for specific drug delivery to the brain (Xue et al., 2021; Jiang et al., 2022). New diagnostic platforms and emerging therapeutic strategies will further develop the engineering and therapeutic potential of small EVs in the coming years.

## 5 Conclusion and perspectives

As carriers of intercellular information exchange, small EVs have shown great promise in preliminary basic laboratory studies for clinical tumor diagnosis and treatment. However, there are some challenges in applying small EVs to clinical applications. The controversy between exosomes and extracellular vesicles has been described in detail in the previous section. In addition to this, the yield of natural small EVs is generally low and difficult to scale up. This creates some difficulties for research and clinical applications. It is unclear whether the resulting engineered small EVs can be used for clinical disease treatment due to their small size, complex components, low yield of isolation and purification, and difficulty in controlling the engineering modification technology.

The most appealing aspect of small EVs in tumor metastasis research is their ability of organotropism and the formation of pre-metastatic ecological niches, and in the future, they hold promise as biomarkers for predicting the occurrence of brain metastasis in patients and for early intervention in such patients to prevent brain metastasis. However, the potential use of small EVs as a disease diagnostic marker is dependent on technological advances in small EVs-based drug delivery systems, and large-scale industrial production of small EVs for clinical therapeutic use faces significant challenges. The precise mechanism of small EVs uptake by target cells, which is critical to the use of small EVs as a drug delivery tool, has yet to be described.

Small EVs’ ability to direct tumor organ propensity and mediate the formation of pre-metastatic ecological niches have provided researchers with new avenues to investigate the mechanisms of breast cancer brain metastasis. Currently, most research has concentrated on pre-metastatic ecological niches and BBB disruption, with only a few laboratories investigating the specific mechanisms by which small EVs promote the proliferation of metastatic tumor cells in the brain microenvironment. The prevention of brain metastasis is the focus of research; however, for the fraction of patients who have already had brain metastasis at the time of breast cancer diagnosis, effective therapeutic approaches are urgently needed. Thus, research on the mechanisms of dynamics and mutual cross-talk between tumor cells and metastatic niches is critical. If applicable, it would be greatly appreciated if relevant studies in this area could be increased. Fortunately, while studying the pathogenesis of breast cancer brain metastasis, some researchers are looking into new ways to treat brain metastases (Sharma et al., 2019; Wei et al., 2021).

In conclusion, investigating tumor cell mechanisms at the gene and protein levels during tumor development is a massive long-term project, but further investigation of the limited information contained in small EVs, as a key pathway of tumor cell information transfer, will undoubtedly provide strong guidance on tumor treatment strategies in the coming years. The ultimate goal is to prevent breast cancer patients without brain metastasis and even other types of tumors from developing, as well as to prolong the overall survival of patients with brain metastasis, and achieve long-term survival with tumors.
